# The complete chloroplast genome of *Phellodendron chinense* (Rutaceae), an Endangered medicinal plant in southern China

**DOI:** 10.1080/23802359.2020.1837688

**Published:** 2020-11-20

**Authors:** Nan Yang, Qiaoyun Liu, Liangcheng Zhao

**Affiliations:** School of Ecology and Nature Conservation, Beijing Forestry University, Beijing, China

**Keywords:** *Phellodendron chinense*, medicinal plant, chloroplast genome, phylogeny

## Abstract

*Phellodendron chinense* is an Endangered medicinal plant in southern China. In this study, the complete chloroplast genome sequence of *P.chinense* was characterized by de novo assembly. The length of the whole chloroplast genome was 158,537 bp, containing a large single copy region (LSC) of 86,250 bp and a small single copy region (SSC) of 18,287 bp, which were separated by a pair of 27,000 bp inverted repeat regions (IRs). The sequence contains 114 unique genes, including 30 tRNA, 4 rRNA, and 80 protein-coding genes. The overall GC content of the chloroplast genome is 38.4% and those in the LSC, SSC, and IR regions are 36.6, 33.2, and 42.9%, respectively. The phylogenetic analysis based on reported chloroplast sequences of Rutaceae showed that *P. chinense* is sister to *P. amurense*, consisting a monophyletic group, and that *Phellodendron* is closely related to *Zanthoxylum*.

*Phellodendron* is a small genus of aromatic deciduous trees in family Rutaceae distributed in eastern Asia. It consists of two recognized species, *P. chinense* Schneid. and *P. amurense* Rupr. (Ma et al. 2006). *Phellodendron chinense* is endemic to Anhui, Hubei, Hunan, Sichuan and Yunnan provinces, southern China, scattering in subtropical broad-leaved forests or mixed forests (Zhang et al. 2019). This species is best known for its bark (cortex) which was used as one of the most famous Chinese traditional medicines (Tang et al. 2016). Due to its high medicinal value, it was listed as one of the 237 species of national Endangered and key protected wild plants in China (Yu 1999). In the past decades, wild populations of *P. chinense* have declined significantly, suggesting that urgent conservation measures need to be taken (Shen et al. 2009; Tang et al. 2016; Zhang et al. 2019). In this study, we assembled the complete chloroplast genome of *P. chinense* using next-generation sequencing to provide a gene source for further genetic and conservation studies.

Fresh young leaves of *P. chinense* were collected from Tianquan County, Sichuan Province, China (N 29°59′19.61ʺ, E 102°38′34.13″). Voucher specimen (collection numbers: BJFUZLC061) was deposited in the Herbarium of Beijing Forestry University (BJFC). The total genome DNA was extracted using a modified protocol (Chen et al. 2014) and sent to Majorbio (http://www.majorbio.com, China) for next-generation sequencing using Illumina Hiseq Xten. About 2.5 Gb high quality, 2 × 150 bp pair-end reads were obtained from High-throughput sequencing. The chloroplast genome of *P. amurense* (Genbank accession no. KY707335.1) was used as a reference to exclude nuclear and mitochondrial reads by Geneious Prime 2020.2 (Kearse et al. 2012). Filtered chloroplast reads were exploited for de novo assembly with Geneious Prime. The assembled chloroplast sequences of *P. chinense* were then annotated using Plann (Huang and Cronk 2015) by referring to the relative group.

The complete chloroplast genome of *P. chinense* (Genbank accession no. MT916287) was a circular molecule with a size of 1,58,537 bp in length, comprising a large single copy (LSC) region of 86,250 bp and a small single copy (SSC) region of 18,287 bp, which were separated by a pair of 27,000 bp inverted repeat (IR) regions. The chloroplast genome sequence contains unique 114 genes. Among them, there are 80 protein-coding genes, 30 tRNA genes and 4 rRNA genes. Most of the genes occurred in the single-copy region, with 4 rRNA genes (*rrn16*, *rrn4.5*, *rrn23*, and *rrn5*), 7 tRNA genes (*trnI-CAU, trnL-CAA, trnV-GAC, trnI-GAU, trnA-UGC, trnR-ACG,* and *trnN-GUU*) and 8 protein-coding genes (*rpl2*, *rpl22*, *rpl23*, *rps7, ycf15*, *ycf2*, *ndhB*, and *rps12*) replicating in the IR region. Among these genes, 16 genes (*rpl2, rpl16, rps12, rps16, ndhB, ndhA, atpF, rpoC1, petB, petD, trnI-GAU, trnK-UUU, trnG-UCC, trnL-UAA, trnV-UAC,* and *trnA-UGC*) had one intron, 2 genes (*ycf3* and *clpP*) contained 2 introns. The overall GC content of the chloroplast genome is 38.4% and those in the LSC, SSC, and IR regions are 36.6, 33.2, and 42.9%, respectively.

In order to understand the phylogenetic status of *P. chinense* within Rutaceae, a phylogenetic analysis was performed based on complete chloroplast genome sequences of 18 other species of the family. These sequences were obtained from NCBI and *Canarium album* (Burseraceae) was used as the outgroup. All sequences were aligned, and a phylogenetic tree was constructed using the maximum likelihood method by MAGE-X (Kumar et al. 2018; Katoh et al. 2019). Bootstraps were calculated for 1000 replicates to comfirm the bootstrap value of each node, and the Tamura-Ne model operation was selected to obtain the tree (Figure 1). The phylogenetic results showed that *P. chinense* is sister to *P. amurense*, consisting a monophyletic group. Then *Phellodendron* is closely related to *Zanthoxylum*, which is consistent with the phylogenetic relationship between the two genera based on plastid and nuclear markers (Poon et al. 2007; Appelhans et al. 2018).

**Figure 1.**
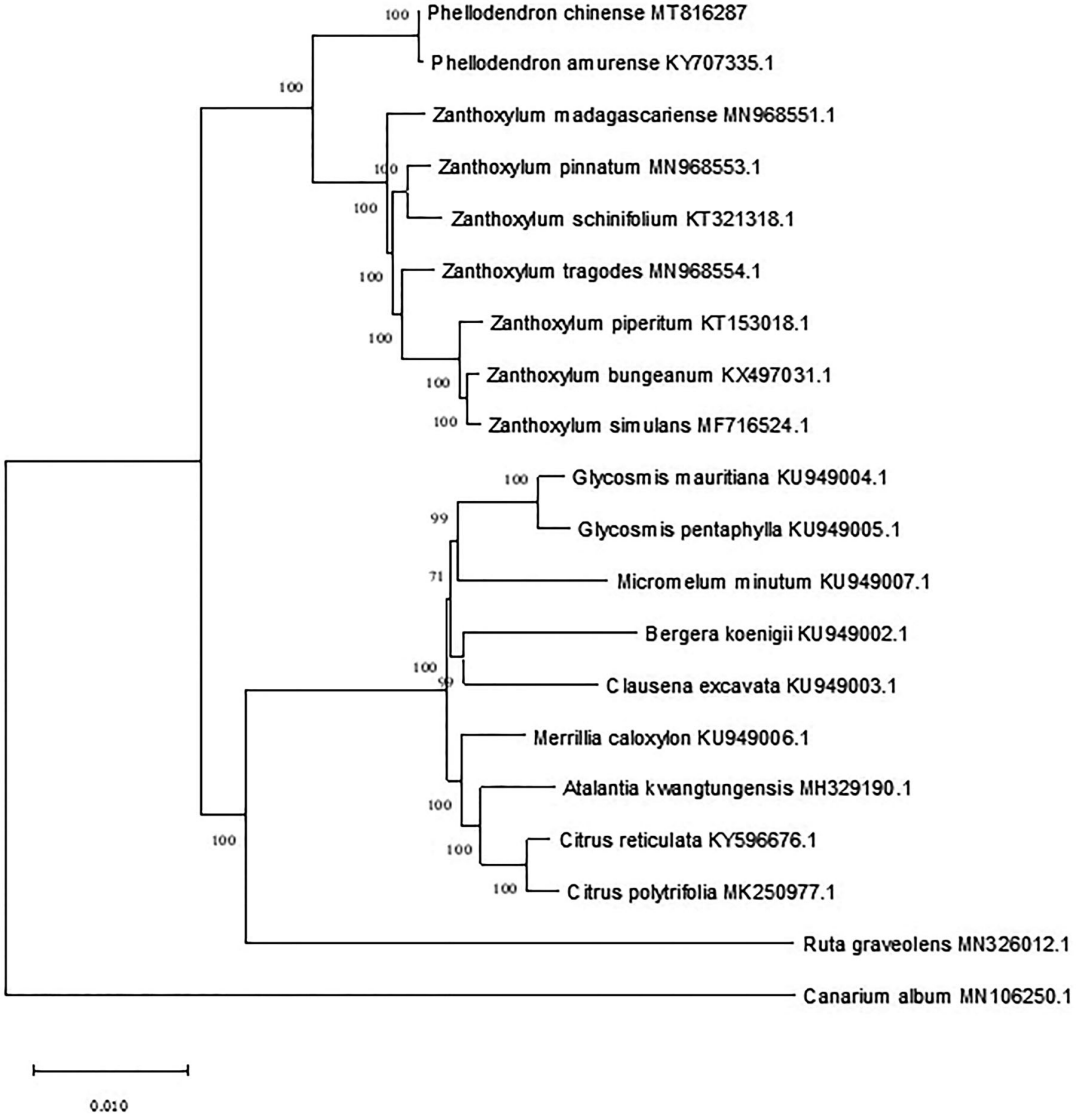
Maximum likelihood tree of 19 Rutaceae species based on the complete plastid genome sequences. The number associated with the nodes are the bootstrap values.

## Data Availability

The data that support the findings of this study are openly available in GenBank of NCBI at https://www.ncbi.nlm.nih.gov, reference number MT916287.
